# The small-nucleolar RNAs commonly used for microRNA normalisation correlate with tumour pathology and prognosis

**DOI:** 10.1038/sj.bjc.6606076

**Published:** 2011-03-15

**Authors:** H E Gee, F M Buffa, C Camps, A Ramachandran, R Leek, M Taylor, M Patil, H Sheldon, G Betts, J Homer, C West, J Ragoussis, A L Harris

**Affiliations:** 1 Molecular Oncology Laboratories, Department of Oncology, University of Oxford, Weatherall Institute of Molecular Medicine, John Radcliffe Hospital, Oxford OX3 9DS, UK; 2 Genomics Laboratory, Wellcome Trust Centre for Human Genetics, Roosevelt Drive, Oxford OX3 7BN, UK; 3 Academic Radiation Oncology, School of Cancer and Imaging Sciences, Christie Hospital NHS Trust, Wilmslow Road, Manchester M20 4BX, UK; 4 Academic Department of Otolaryngology-Head and Neck Surgery, Manchester Royal Infirmary, Manchester, UK

**Keywords:** snoRNA, microRNA, normalisation, prognosis

## Abstract

**Background:**

To investigate small-nucleolar RNAs (snoRNAs) as reference genes when measuring miRNA expression in tumour samples, given emerging evidence for their role in cancer.

**Methods:**

Four snoRNAs, commonly used for normalisation, RNU44, RNU48, RNU43 and RNU6B, and miRNA known to be associated with pathological factors, were measured by real-time polymerase chain reaction in two patient series: 219 breast cancer and 46 head and neck squamous cell carcinoma (HNSCC). SnoRNA and miRNA were then correlated with clinicopathological features and prognosis.

**Results:**

Small-nucleolar RNA expression was as variable as miRNA expression (miR-21, miR-210, miR-10b). Normalising miRNA PCR expression data to these recommended snoRNAs introduced bias in associations between miRNA and pathology or outcome. Low snoRNA expression correlated with markers of aggressive pathology. Low levels of RNU44 were associated with a poor prognosis. RNU44 is an intronic gene in a cluster of highly conserved snoRNAs in the growth arrest specific 5 (GAS5) transcript, which is normally upregulated to arrest cell growth under stress. Low-tumour GAS5 expression was associated with a poor prognosis. RNU48 and RNU43 were also identified as intronic snoRNAs within genes that are dysregulated in cancer.

**Conclusion:**

Small-nucleolar RNAs are important in cancer prognosis, and their use as reference genes can introduce bias when determining miRNA expression.

There is increasing interest in measuring the levels of microRNAs (miRNAs) in tumours using real-time polymerase chain reaction (RT–PCR) methods as markers of pathology and prognosis (Catto *et al*, 2009; Hummel *et al*, 2010). Real time polymerase chain reaction determines the relative expression of variably expressed target miRNAs in comparison with one or more stably expressed reference genes (also called housekeeping or internal control genes). This normalisation is required to allow for variability in miRNA quantity and/or cDNA synthesis. However, uncertainty remains over the normalisation process and selection of appropriate reference genes (Hui *et al*, 2009; Chang *et al*, 2010), and their impact on results obtained (Gee *et al*, 2008; Peltier *et al*, 2008). This uncertainty has implications for the development of clinically useful miRNA signatures, as it does for gene expression signatures such as Oncotype Dx (Kaklamani, 2006; van den Broek *et al*, 2009).

The current convention for miRNA RT–PCR is to normalise to one reference gene (usually RNU6B, RNU44 or RNU48) (Roa *et al*, 2010). These small-nucleolar RNAs (snoRNAs), non-protein coding RNA, are approximately 70 nucleotides in length, and involved in processes such as site-specific modification of nucleotides in target RNAs (Mattick and Makunin, 2005). The snoRNAs RNU48, RNU44 and RNU43 are members of the large C/D box family, thought to direct 2′-*O*-ribose methylation in ribosome biogenesis (Kiss, 2002). RNU6B (U6) is part of the U6 small-nuclear ribonucleoprotein, a component of the spliceosome upon which splicing of pre-mRNA occurs. The Applied Biosystems Megaplex miRNA assay pool (Applied Biosystems, Warrington, UK) and associated RT–PCR arrays, such as Applied Biosystems Taqman Low-Density Arrays (Applied Biosystems), recommend using the average of RNU44 and RNU48 (with U6 additionally in the latest version) (Product Information for TaqMan Array Gene Signature Cards, 2010; Hui *et al*, 2010).

Although the function of snoRNAs is still poorly understood, recent evidence suggests they are deregulated in cancer. The non-coding growth arrest specific transcript 5 gene (*GAS5*), which encodes multiple snoRNAs, induces growth arrest and apoptosis in breast cancer cell lines, and is significantly downregulated in breast cancer (Mourtada-Maarabouni *et al*, 2009). Growth arrest specific transcript 5 gene appears to suppress transcriptional activity induced by glucocorticoid receptors by inhibiting the binding of receptors to glucocorticoid response elements (Kino *et al*, 2010). Another snoRNA, U50, is involved in the development of prostate and breast cancer, although its function is unknown (Dong *et al*, 2009).

Given the emerging evidence for a role of snoRNAs in cancer, the aim of the work reported here was to investigate the appropriateness of their use as reference genes when measuring miRNA expression in tumour samples. Tumour expression of the snoRNAs RNU44, RNU48, RNU43 and U6 was studied in relation to clinicopathological features and prognosis, and as reference genes for normalisation of miRNA expression data. The work was carried out in two series of patients: 219 breast cancers and 46 head and neck squamous cell carcinoma (HNSCC).

## Materials and methods

### Clinical samples

Ethical approval was obtained from the local research Ethics Committees (Oxford and South Manchester). The breast cancer series consisted of 219 patients with early-first primary breast cancer, treated in Oxford between 1989 and 1992. Patients received surgery followed by adjuvant treatment. The data set was complete for age, nodal status, definitive surgery, relapse and survival. The patient demographics and details of treatments given are provided in (Camps *et al*, 2008) and Supplementary Table 1. The HNSCC series comprised of 46 patients with primary HNSCC. Full-patient demographics and treatment details are provided in (Gee *et al*, 2010) and Supplementary Table 1. All patients underwent surgical resection with curative intent, and post-operative radiotherapy was given to all but five patients.

### RNA extraction

Patient tumour samples taken at operation were placed in RNAlater (Applied Biosystems) for up to 12 h before cryopreservation in liquid nitrogen. Subsequently, the samples were divided and half paraffin embedded for histological analysis. The RNA was extracted from the remaining tissue using Tri Reagent (Sigma-Aldrich, Poole, UK). Quality and quantity of RNA were confirmed using the NanoDrop ND-1000 spectrophotometer and the Agilent 2100 Bioanalyzer (Agilent Technologies, Santa Clara, CA, USA). Cases were excluded in which tumour was present in <10% of a representative hematoxylin and eosin section.

### Real time-reverse transcription PCR

Expression of miR-210, miR-21, miR-10b, miR-342 and miR-30a-3p, and three snoRNAs, RNU43, RNU44 and RNU48 were measured by RT–PCR according to the TaqMan MicroRNA Assay protocol (Applied Biosystems) in the entire breast cancer series. U6 was also measured on a subset of 48 of the 219 breast cancer cases, chosen as they were the most representative of the various molecular subtypes of breast cancer (e.g., Luminal A). The expression of miR-210, miR-21, miR-10b, RNU43, RNU44 and RNU48 was measured in the HNSCC series. Complementary DNA was synthesised from 5 ng of total RNA using TaqMan miRNA primers and the TaqMan MicroRNA Reverse Transcription Kit (both from Applied Biosystems). Real-time polymerase chain reaction was carried out in triplicate as described previously (Camps *et al*, 2008). Fold changes in miRNA expression were determined by the 2-ΔΔC_t_ method. In which discussed, results were also normalised using RNU43, RNU44, RNU48 and U6 (for the subset of breast cases), the average of two snoRNAs or the median of three snoRNAs.

### Immunohistochemistry

Immunohistochemistry for HIF-1*α* was performed on sections from formalin-fixed, paraffin-embedded tumour biopsy samples. Details of antibodies and scoring system are given elsewhere (Beasley *et al*, 2002).

### Microarray data

A total of 72 of the 219 samples in the breast cancer series and the 46 HNSCC samples were also expression profiled using Affymetrix GeneChips (Santa Clara, CA, USA), with standard pre-processing and normalisation (Winter *et al*, 2007; Loi *et al*, 2008). To assess relationship between probe sets to GAS5 and outcome in breast cancer, a large breast cancer series was also used (*n*=152, Loi *et al*, 2008), which overlapped by 72 cases with the Camps series of 219 (details in Supplementary Table 1). Published metagene signatures were used: to determine molecular classification of subtype (Sorlie *et al*, 2001); to measure hypoxia (Winter *et al*, 2007; Buffa *et al*, 2010); for proliferation, invasion and immune response scores (Desmedt *et al*, 2008); and for ErBB2 scores (genes in common between (Desmedt *et al*, 2008) and (Wirapati *et al*, 2008)). For the 219 breast cases, miRNA expression profiles were also measured using 200 ng of total RNA on Illumina (San Diego, CA, USA) miRNA arrays version 1 as per the manufacturer's instructions. Average signal was background subtracted with local background subtraction (BeadStudio, Illumina), quantile normalised and logged (base2) in R.6 (http://www.r-project.org/).

### Gene mapping

Small-nucleolar RNAs were mapped to genomic regions using the gene accession numbers and the UCSC browser. Publically available data sets were accessed using Oncomine (http://www.oncomine.org).

### Statistical methods

The gene stability measure (*M*), mean pairwise variation for a gene compared with all other tested reference genes, was derived from the GeNorm algorithm (Vandesompele *et al*, 2002) and the SLqPCR package (http://www.bioconductor.org). Unless otherwise stated, correlations of snoRNAs and miRNAs, with clinical and pathological variables, were assessed using the appropriate non-parametric methods (Spearman's test for continuous variables, Wilcoxon, Kruskal–Wallis or Mann–Whitney's *U*-test for categorical variables). Correlation between snoRNA and probe sets was performed using Pearson correlations. Tumour recurrence and death were calculated from the time of surgery, for the breast series according to STEEP criteria (Hudis *et al*, 2007) or as previously defined by us (Camps *et al*, 2008). Univariate survival analysis was carried out by applying the log-rank test to miRNA or snoRNA expression levels stratified by median values. Disease-specific overall survival (called overall survival in the rest of this article), recurrence-free survival (RFS) and distant RFS (DRFS) were considered as outcomes. Where Cox survival analyses were used, the fractional rank of the miRNA or snoRNAs was considered: the patients were ranked using expression levels and the ranks were normalised between 0 and 1. SPSS 17 (SPSS Inc., Chicago, IL, USA) and R.6 (http://www.r-project.org) were used for statistical analyses.

## Results

### SnoRNAs, commonly used as reference genes, show high variability of expression in cancer

**Figure 1:**
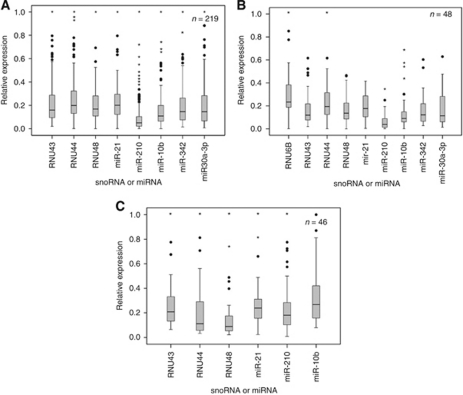
Values of snoRNAs vary as widely as miRNAs. Box and whisker plots of snoRNA and miRNA expression in the 219 breast cancer cases (**A**), a subset of 48 cases, chosen as most representative of the various molecular subtypes of breast cancer (**B**), and the HNSCC series (**C**). For all plots, relative expression measured by RT–PCR: shown as median (line) and interquartile range (box), outliers (circles) and extreme cases (stars).

We first noted that the expression of snoRNAs was highly variable in cancer, with similar ranges of values to some of the most varying miRNAs (Figure 1). In the 219 breast cancer cases, miR-21, which has been found in multiple studies to be a prognostic factor in breast cancer (Yan *et al*, 2008), had a median value of 0.20, an interquartile range of 0.18 and a s.d. of 0.14. Similarly, RNU44 had a median value of 0.20, an interquartile range of 0.20 and a s.d. of 0.17. This wide range of values was even more striking in the subset of 48 cases (Figure 1B), in which U6 had also been measured. These 48 cases were chosen for further analysis as they were the most representative of the different breast cancer molecular subtypes (Simpson *et al*, 2010), and are therefore genetically relatively distinct from each other. This can be more formally measured by the *M* values, a measure of the mean pairwise variation for a gene compared with all other tested control genes (higher number representing greater noise/variability). We found in the breast series that RNU43 RNU44 and RNU48 had *M* value as 1.32, 1.22 and 1.27, respectively. The values for the 48 breast cancer subset were 1.59, 1.42 and 1.76, respectively. The corresponding values for the 46 HNSCC were 0.70, 0.72 and 0.62, respectively.

### SnoRNAs used as reference genes introduce bias

For each miRNA studied, there was a tighter correlation between the raw and normalised values when measured by microarray than by RT–PCR (Supplementary Table 2 and Supplementary Figure 1). For example, for miR-210, the Spearman's correlation coefficient (*ρ*) was 0.94 when comparing raw and normalised microarray breast cancer data, whereas it was 0.89 for raw and normalised RT–PCR data (*P*<0.001 for both) (Supplementary Figure 1). Concerningly, given many papers have been published on miR-21, the corresponding *ρ* values of miR-21 were 0.24 for microarray and 0.34 for RT–PCR, suggesting there is significant variability with probe or primer variants.

**Figure 2:**
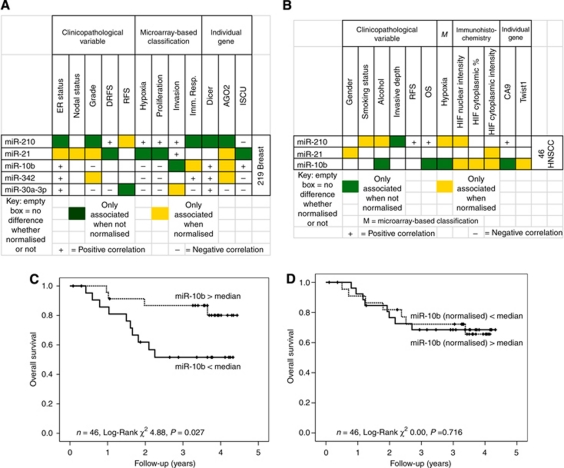
Normalising miRNA expression data to snoRNAs introduces bias. Association between miRNA and clinicopathological factors can be over- or underestimated, depending on the interaction with snoRNA control. MicroRNA and snoRNA measured by RT–PCR in 219 breast cancers (**A**) and 46 HNSCCs (**B**). Background colour of box indicates type of misassociation: green boxes – miRNA associated with factor before it is normalised to snoRNA; yellow boxes – miRNA not associated until normalised; white boxes – consistent association between miRNA and factor whether normalised or not; ±show direction of statistically significant correlation (if any). ER status: oestrogen receptor status; nodal status: binary; smoking/alcohol status: never, ex-user, current user. DRFS or RFS: distant recurrence-free survival or recurrence-free survival (STEEP criteria), OS, overall survival; expression stratified by median value, positive correlation, higher level associated with poorer prognosis. Details of microarray-based classifications, including hypoxia metagene score, proliferation score, invasion score, immune response score (Imm. Resp.) and scoring system for immunohistochemistry, in methods section. AGO2, EIF2C2, (Argonaute 2), ISCU, Iron-Sulphur Cluster Homologue (*Escherichia coli*), CA9, carbonic anhydrase 9; (**C** and **D**) Kaplan–Meier curves for overall survival for patients with HNSCC according to expression of miR-10b measured by RT–PCR, stratified by median value. (**C**) shows relative expression of miR-10b only, (**D**) shows miR-10b normalised to three control genes. The colour reproduction of this figure is available on the html full text version of the manuscript.

For the five miRNA measured in the 219 breast cancer cases and the three miRNA measured in the 46 HNSCC cases, contrasting results were obtained for normalisation. Normalisation could either unmask or mask associations between miRNA expression and clinicopathological factors (Figure 2). The former type of problem occurred when the miRNA was not associated with a given clinicopathological factor until it was normalised to the control gene(s). The latter occurred when the miRNA was associated with a given clinicopathological factor only before it was normalised. These misassociations are shown graphically in Figure 2A (219 breast cases) and Figure 2B (46 HNSCC cases), and with *P*-values in Supplementary Figures 3A and B. This finding is further illustrated for miR-10b in the HNSCC series in which normalisation to the three reference genes (which trend down with poor prognosis, as does miR-10b) abrogated the association of unnormalised miR-10b levels with prognosis (Figure 2, panels C–D, *P*-values 0.027 and 0.716, for raw and normalised data, respectively). The opposite effect (when normalisation enhanced significance) was observed for miR-210 in the breast cancer cases, which goes up in association with a poor prognosis. The *P*-values for miR-210 and RFS were 0.01 and 0.003 for raw and normalised data, respectively, data not shown.

### snoRNA expression is associated with clinicopathological factors

**Figure 3:**
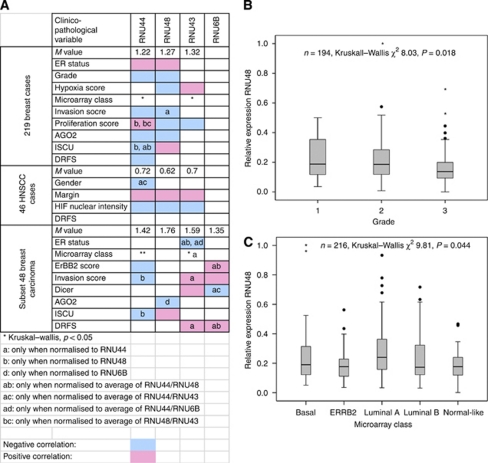
Association between snoRNAs and clinicopathological factors. (**A**) Heat map showing direct association or correlation between snoRNAs (measured by RT–PCR) and clinicopathological factors. Blue background – negative correlation; pink background – positive correlation; white background – no correlation, except ^*^ (significant association with molecular subtype). *M* value – gene stability measure, defined as average pairwise variation of a particular gene with all other control genes, measured by geNorm algorithm. ER, oestrogen receptor; AGO2, EIF2C2, Argonaute 2; ISCU, Iron-Sulphur Cluster Homologue (*E. coli*); DRFS, distant recurrence-free survival (STEEP criteria), Cox regression. Proliferation, ErBB2, invasion scores derived from microarray; HIF nuclear scores from immunohistochemistry, details in Materials and Methods section. Results shown for Spearman correlation (continuous variables), Mann–Whitney (two categorical variables), Kruskal–Wallis (>2 categorical variables). (**B**) RNU48 decreases significantly with histological grade (Kruskal–Wallis). (**C**) RNU44 varies across molecular subtype of breast cancer (Kruskal–Wallis). For panels B–C, results show relative expression measured by RT–PCR in the breast cancer series (where available): shown as median (line) and interquartile range (box), outliers (circles) and extreme cases (stars). The colour reproduction of this figure is available on the html full text version of the manuscript.

To understand why these snoRNAs reference genes may introduce bias, we looked at their relationship with clinicopathological factors, gene expression signatures of biological phenotypes and the expression of miRNA processing genes (*Dicer* and *Argonaute 2*, henceforth called AGO2). The uncorrected snoRNAs were directly associated with multiple factors, including oestrogen receptor (ER) status, grade, microarray-based markers of proliferation and invasion, and miRNA processing genes (Figure 3A, and with *P*-values in Supplementary Figure 3C). For example, RNU48 was negatively correlated with tumour grade (Figure 3B). The miRNA varied significantly across molecular subtype classification (data available for 216 of 219 cases), with higher levels associated with Luminal A tumours: RNU43 *P*-value 0.023; RNU44 *P*-value 0.044. Figure 3C shows the results for one representative snoRNA. In addition, we studied the expression of snoRNAs when normalised to the other snoRNAs. We noted many examples of potential bias introduced by the other snoRNAs. For example, RNU44 was positively correlated with proliferation score only when normalised to RNU48 or to the average of RNU48 and RNU43. As RNU48 and RNU43 were negatively correlated with proliferation score, this suggested normalising to them introduced bias.

### RNU44 expression is associated with survival

**Figure 4:**
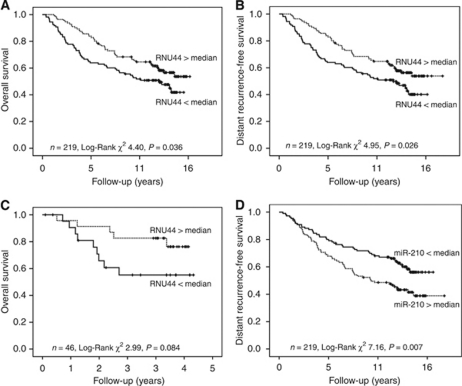
RNU44 is prognostic in HNSCC and breast cancer. (**A**) Overall and (**B**) distant recurrence-free Kaplan–Meier survival curves for patients with breast cancer stratified according to relative expression of RNU44 measured by RT–PCR. (**C**) Kaplan–Meier curve showing overall survival of patients with HNSCC stratified according to relative expression of RNU44 measured by RT–PCR. (**D**) Distant recurrence-free Kaplan–Meier survival curves for patients with breast cancer stratified according to relative expression of miR-210 measured by RT–PCR, provided for comparison (*n*=219). For all four panels, expression levels are stratified by median value.

In 219 patients with breast cancer, lower level of tumour RNU44 expression was an adverse prognostic factor for overall survival and DRFS in univariate analysis when considered as a binary variable divided by median value (Figures 4A and B). Expression of RNU44 was also significant in a Cox regression model (*P*-value 0.049), with a hazard ratio of 0.5 and 95% confidence intervals of 0.26–0.99. This was not significant in multivariate analysis (Supplementary Table 3). In the series of 46 HNSCC, RNU44 lower than the median was again associated with a poor prognosis, but this was not statistically significant, perhaps because of the small numbers of cases available (Figure 4C). A survival curve for miR-210, which we and others (Camps *et al*, 2008) have previously shown to be prognostic in breast and HNSCC, is provided for comparison (Figure 4D).

### RNU44 is an intronic snoRNA within *GAS5*, which is associated with prognosis in HNSCC and breast cancer

**Figure 5:**
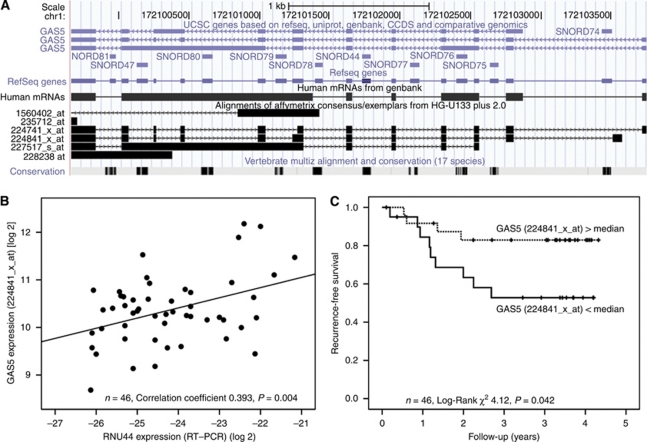
Growth arrest specific 5 and cancer. (**A**) Growth arrest specific 5 contains highly conserved snoRNAs. A screen shot of the USCS Genome Browser shows multiple snoRNAs contained within introns of *GAS5*, which are highly conserved across species. Affymetrix probe sets to *GAS5* are also shown. (**B**) Correlation between RNU44 measured by RT–PCR and *GAS5* expression value in Affymetrix microarray, results for one representative probe shown. (**C**) Growth arrest specific 5 is a marker of prognosis in HNSCC. Kaplan–Meier curve of RFS for patients with HNSCC stratified according to relative expression of *GAS5* measured by microarray. Expression levels are stratified by median value.

To elucidate the mechanism by which the three snoRNAs were associated with pathological factors and survival, we mapped the snoRNAs to their genomic location. RNU44 mapped to an intronic region of *Homo sapiens* growth arrest specific 5 non-protein coding RNA (GAS5, RefSeq NR_002578.2). The *GAS5* transcript, which contains a cluster of highly conserved snoRNAs, can be interrogated with multiple probes available on Affymetrix arrays (Figure 5A). We found a correlation between several of these probe sets and the relative expression of RNU44, measured by RT–PCR (Table 1, and Figure 5B, one representative probe set shown).

Growth arrest specific 5 has previously been reported to be downregulated in breast cancer compared with normal tissue (Mourtada-Maarabouni *et al*, 2009); however, its role in prognosis or in other cancers has not been elucidated. We examined its expression in publically available data sets using Oncomine. Growth arrest specific 5 was downregulated by 2.9-fold (*P*-value 6.21 × 10^−7^) in a microarray of 22 glioblastoma multiforme compared with normal brain samples (Lee *et al*, 2006).

In our series of HNSCC, when expression values for the probes to *GAS5* were explored as prognostic markers, a trend similar to the one seen for RNU44 was observed in Kaplan–Meier analyses (Table 1 and Figure 5C, one representative probe shown). Specifically, a high level of *GAS5* was associated with a good prognosis. At the conclusion of follow-up, only 55% of patients with *GAS5* less than median were alive, whereas 84% of patients with *GAS5* higher than median were still alive (probe set 224841_x_at, *P*-value 0.042). Results for the other three probe sets are shown in Supplementary Figure 2. Of note, only the probe sets with a strong correlation to RNU44 were significantly associated with prognosis. Owing to the small size of the series, it was not possible to perform multivariate analysis.

Next, we examined the subset of the series of 219 patients with breast cancer for which Affymetrix gene expression data were available (the overlapping series of 152 cases). A similar trend was observed with lower levels of GAS5 being associated with a poor prognosis but this was not statistically significant (Table 1).

### RNU43 and RNU48 are also intronic snoRNAs within genes that are associated with cancer

RNU43, which was lower in tumours with a poorer prognosis (although not statistically significantly), mapped to an intronic region of *H. sapiens* ribosomal protein L3 (*RPL3*, RefSeq NM_001033853.1 and NM_000967.3). Alternate transcriptional splice variants, encoding different isoforms, have been characterised. *RPL3* belongs to the L3P family of ribosomal proteins and encodes a ribosomal protein that is a component of the 60S subunit. In publically available data sets, interrogated using Oncomine, *RPL3* was downregulated: two-fold in ovarian adenocarcinoma compared with normal ovary (*P*-value 6.94 × 10^−10^) (Welsh *et al*, 2001), and 36-fold in breast tumour stroma compared with normal stroma (*P*-value 1.06 × 10^−28^) (Finak *et al*, 2008).

When mapped to Affymetrix probe sets (Supplementary Table 4) in our series, the results were complex. In the HNSCC series, there was a good correlation between several probe sets and RNU43 measured by RT–PCR, but *RPL3* was not associated with prognosis. In the breast series, there was a poor correlation between *RPL3* and RNU43, but low levels of RPL3 were associated with a poor prognosis in this series.

RNU48 is also an intronic snoRNA, within chromosome 6 open reading frame 48 (c6orf48 or G8). C6orf48 is part of the major histocompatability complex III tissue-protective factor produced in response to chronic inflammation, and polymorphisms in the *c6orf48* gene are associated with susceptibility to infection (Kerr *et al*, 2005). Several Affymetrix probe sets map to the region containing c6orf48: 220755_s_at and 222968_at. Only 222968_at correlated with RNU48 by RT–PCR but in a negative direction (Spearman's *ρ* −0.344, *P* value 0.012), suggesting that these probe sets are not a helpful way of interrogating RNU48 values.

### RNU6B may be associated with fewer clinicopathological factors than the other snoRNAs

U6 was not directly associated with clinicopathological factors, with the exception of invasion gene expression signature score. It was associated with several factors when normalised to the average of other snoRNAs, suggesting that these were introducing bias into the results (Figure 3A). We were only able to test the use of four control genes in the small subset of 48 cases. In this small series, normalising to four genes abrogated the statistically significant association of miR-210 or miR-30a-3p with prognosis, suggesting that noise was introduced. However, normalising to U6 alone gave as good as, or better significance, than normalising to multiple genes, suggesting that U6 is a more stable reference gene than the other snoRNAs tested (Supplementary Table 5).

## Discussion

Normalisation of miRNA data measured by RT–PCR is critical to interpreting clinical significance and developing miRNA as tumour markers. Our work highlights several key problems in the way this has been carried out, and may explain many opposing or non-confirmatory studies in this area. The first issue we noted was that snoRNAs had highly variable expression and introduced noise when used as controls. For microarray data, there was a good internal correlation between quantile normalised and non-normalised data for a given miRNA, however, the internal correlation between the relative expression by RT–PCR of miR-210, and miR-210, normalised to three control genes, was noisier. This is likely to be because of the robustness of standardisation of gene array data, with thousands of probes contributing to normalisation of arrays and well-validated methodology. miR-210 is among the miRNAs most reproducibly associated with outcome, is highly expressed and has a wide range of expression levels, but the above issue will be even more important in the case of less varying and lower expressed miRNAs.

Indeed, the noise introduced by normalising to snoRNAs may explain why RT–PCR has been questioned as the ‘gold standard’ for validating miRNA results by Git *et al* (2010), who found in a comparison of multiple platforms and RT–PCR that false-positive calls were more likely to be generated by RT–PCR than by microarray. We noted that expression of snoRNAs varied as much as miR-21, which has been associated with prognosis in multiple studies (Markou *et al*, 2008; Yan *et al*, 2008). The *M* values for the snoRNAs were higher than expected: for example, in a study of multiple mRNA housekeeping genes, Vandesompele *et al* (2002) typically found when three housekeeping genes were measured that *M* values were less than 0.5 . This may be because of biological differences, such as the existence of multiple subtypes of breast cancer, with different intrinsic snoRNA levels, and variation among samples induced by ER and HER2 status, the heterogeneity in HNSCC site subtypes and differences in relative amounts of tumour and stromal tissue between samples. In addition, it may relate to sample collection methods. The sample collection for HNSCC was carried out by clinical fellows in the operating theatre to a highly standardised protocol, while for the breast samples it was performed according to standard surgical procedure. Although this may have contributed to reduced variability in the HNSCC, it did not eliminate the problems of normalisation, and illustrates the pitfalls of attempting to introduce miRNA-based signatures to routine clinical practice.

Raw data are expected to be noisy but normalisation to genes, which also have variable expression, introduces more noise and possibly bias in the results. Normalisation of miR-10b to the three control genes (which also trend down with poor prognosis, albeit not significantly) abrogated the association of miR-10b with a good prognosis, whereas for miR-210, which is a marker of poor prognosis, the opposite effect was observed (i.e., increased the association), although the results were significant whether normalised or not. This suggested that in some cases, use of snoRNAs as reference genes introduces bias as well as noise. For miRNA, with a large expression range and strong signal, such as miR-210, normalisation problems may be less important than for miRNA with a small range or subtle, although important, biological effect (e.g., miR-10b).

This is, to our knowledge, the first study showing association between snoRNAs with multiple factors, including ER status, grade and miRNA processing genes such as *Dicer* and *AGO2*. This implies that regulation of ribosomal RNA cannot be considered independently from, and may interact both with, these biologically important factors, and with the processing of other noncoding RNA such as miRNA by their interaction with *Dicer* and *AGO2*. Of note, the snoRNAs varied significantly across molecular subtype classification, implying the alteration of multiple species of RNA, both ‘coding’ and ‘non-coding’, across the groups. Furthermore, the finding that RNU44 levels were highest in Luminal A type breast tumours may have been expected from the observation that RNU44 is linked to ER status – however, it is interesting that RNU43, which is not linked to ER status, is even more highly correlated with Luminal A tumours. Recently, poor concordance between molecular classification when using different algorithms has been described (i.e., they did not reliably assign the same patients to the same molecular subtypes) (Weigelt *et al*, 2010). The future addition of information about snoRNAs and other small non-coding RNAs may improve the performance of such classifiers.

This is also, to our knowledge, the first study showing an association between snoRNAs, such as RNU44, and prognosis in both breast and HNSCC. The association between low expression of RNU44 and poor prognosis is in keeping with the study of Mourtada-Maarabouni *et al* (2009), showing that GAS5 sensitises cells to apoptosis in response to stress. Cells which fail to express *GAS5* will evade apoptosis in response to the stressful stimuli, which a tumour cell would experience in a poorly vascularised microenvironment, intermittently depleted of nutrients and oxygen. Mourtada-Maarabouni *et al* (2009) attempted to elucidate the smallest possible area required for apoptosis induction, but their focus was on exons rather than introns (such as RNU44). However, as shown in Figure 5A, it is only the intronic snoRNA that are highly conserved through evolution, suggesting these are the main functional components of GAS5. Furthermore, it is the first time that low GAS5 expression has been shown to be a marker of poor prognosis in HNSCC (and breast).

Where there was a good correlation between probe set and intronic gene (e.g., 224741_x_at and 224841_x_at for RNU44), these microarray data can be mined for prognostic association, however, where there is a poor correlation (e.g., 227517_x_at or 228238_x_at), data mining was not possible (Table 1, Supplementary Figure 2). Indeed, Figure 5A shows that the probe set 228238_x_at would be too short to cover the area of GAS5-containing RNU44.

This was reinforced by the complex relationship between RNU43 and RPL3. In many cases, introns containing snoRNAs occur within protein-coding transcripts, encoding ribosomal proteins, and others involved in ribosomal biogenesis, and RNU43 would initially appear to be an example of this type of parallel genetic output. However, the mapping of RNU43 to RPL3-linked Affymetrix probe sets has not shown a good link between RPL3 and RNU43, so further work is required to elucidate the reason for the difference (for example trough differential regulation of a snoRNA to its host gene, or through alternative splicing, as there are several transcript variants giving rise to different isoforms of RPL3).

U6, highly conserved across evolution, has multiple copies in the genome, and appears to be upregulated in cervical cancer and correlate with progression (Hansen *et al*, 2009). Of note, when miRNA were normalised to U6, there were fewer errors in association between miRNA and clinicopathological factors, and fewer direct associations between U6 and these factors (Figure 3A).

Taken together, these data suggest that RNU44, commonly used for normalisation of miRNA (especially as it appears in the Applied Biosystems Megaplex Pool and Low-Density miRNA array), is a poor choice of normalisation gene. U6 appears the most stable of the snoRNAs tested in breast cancer. Caution should be exercised in using RNU43 and RNU48. RNU43, RNU44 and RNU48 were recommended by manufacturers, because of their stability across multiple normal tissue types (Wong *et al*, 2007), rather than their stability between normal compared with tumour tissue, or different grades of tumour.

The reason for the association between these snoRNAs and clinicopathological factors is not known at present. Many occur in polycistronic clusters, or in imprinted regions, suggesting that they are specifically regulated and in turn have specific regulatory roles in the differential modification of selected target RNAs and in the synthesis of ribosomal proteins (Mattick and Makunin, 2005).

Ribosomal DNA occurs at many known recombination hot spots (Stults *et al*, 2009) and ribosomal proteins are known to be dysregulated in cancer (van Riggelen *et al*, 2010), but little is known about how this occurs, or the role of their intronic snoRNAs. We noted generally lower levels of the snoRNAs in association with poor prognosis or more aggressive tumours. A decrease in ribosomal protein biogenesis may contribute to chromosomal instability, a hallmark of cancer. For example, depletion of RPL3 (and other members of the Yph1 complex) resulted in an increase in abnormal mitoses and aberrant metaphase plates (Killian *et al*, 2004). Additionally, many chemotherapeutic agents inhibit the biogenesis of ribosomes (Killian *et al*, 2004), so downregulating these proteins may evade chemotherapy.

In summary, the clear dysregulation of multiple snoRNAs and their host genes in cancer suggests a novel area of research in cancer initiation and progression. Their use as reference genes can introduce bias when determining miRNA expression, which is reduced by the use of U6, and by validating tissue-specific control genes in relevant cancer (rather than normal) samples.

## Figures and Tables

**Table 1:** Correlation between RNU44 and Affymetrix probes to GAS5

**Affymetrix probe set**	**Value**	**RNU44 (RT–PCR)**	**Recurrence-free survival**	**224741_x_at**	**224821_x_at**	**227517_x_at**	**228238_s_at**
*Series*							
*HNSCC* (n=*46*)							
224741_x_at	CC	0.392		n/a	0.992	0.491	0.57
	*P*	*0.004* ^**^	*0.042*		<0.001	<0.001	<0.001
224841_x_at	CC	0.402		0.992	n/a	0.499	0.578
	*P*	*0.002* ^**^	*0.042*	<*0.001*		<0.001	<0.001
227517_x_at	CC	0.332		0.491	0.499	n/a	0.833
	*P*	*0.017* ^*^	0.937	<0.001	<0.001		<0.001
228238_s_at	CC	0.238		0.571	0.578	0.833	n/a
	*P*	0.092	0.933	<0.001	<0.001	<0.001	
							
*Breast* (n=*152*)^a^							
224741_x_at	CC	0.452		n/a	0.987	0.358	0.466
	*P*	<0.001^**^	0.073		<0.001	<0.001	<0.001
224841_x_at	CC	0.443		0.987	n/a	0.381	0.458
	*P*	<0.001^**^	0.115	<0.001		<0.001	<0.001
227517_x_at	CC	0.305		0.358	0.381	n/a	0.882
	*P*	*0.009* ^**^	0.962	<0.001	<0.001		<0.001
228238_s_at	CC	0.305		0.446	0.458	0.882	n/a
	*P*	*0.009* ^**^	0.852	<0.001	<0.001	<0.001	

Abbreviations: CC=correlation coefficient; n/a=not available; *P*=two-tailed significance; RT–PCR=real-time polymerase chain reaction.

a Loi series – overlap of 72 cases with Camps series. The values shown in italics are statistically significant (*P*-values <0.05). ^*^P<0.05, ^**^P<0.01, ^***^P<0.001.
