# Supplementary figures and images for: The small-nucleolar RNAs commonly used for microRNA normalisation correlate with tumour pathology and prognosis
Source: Br J Cancer. 2011 Mar 15;104(7):1168–77. doi: 10.1038/sj.bjc.6606076 (PMC3068486; doi:10.1038/sj.bjc.6606076)

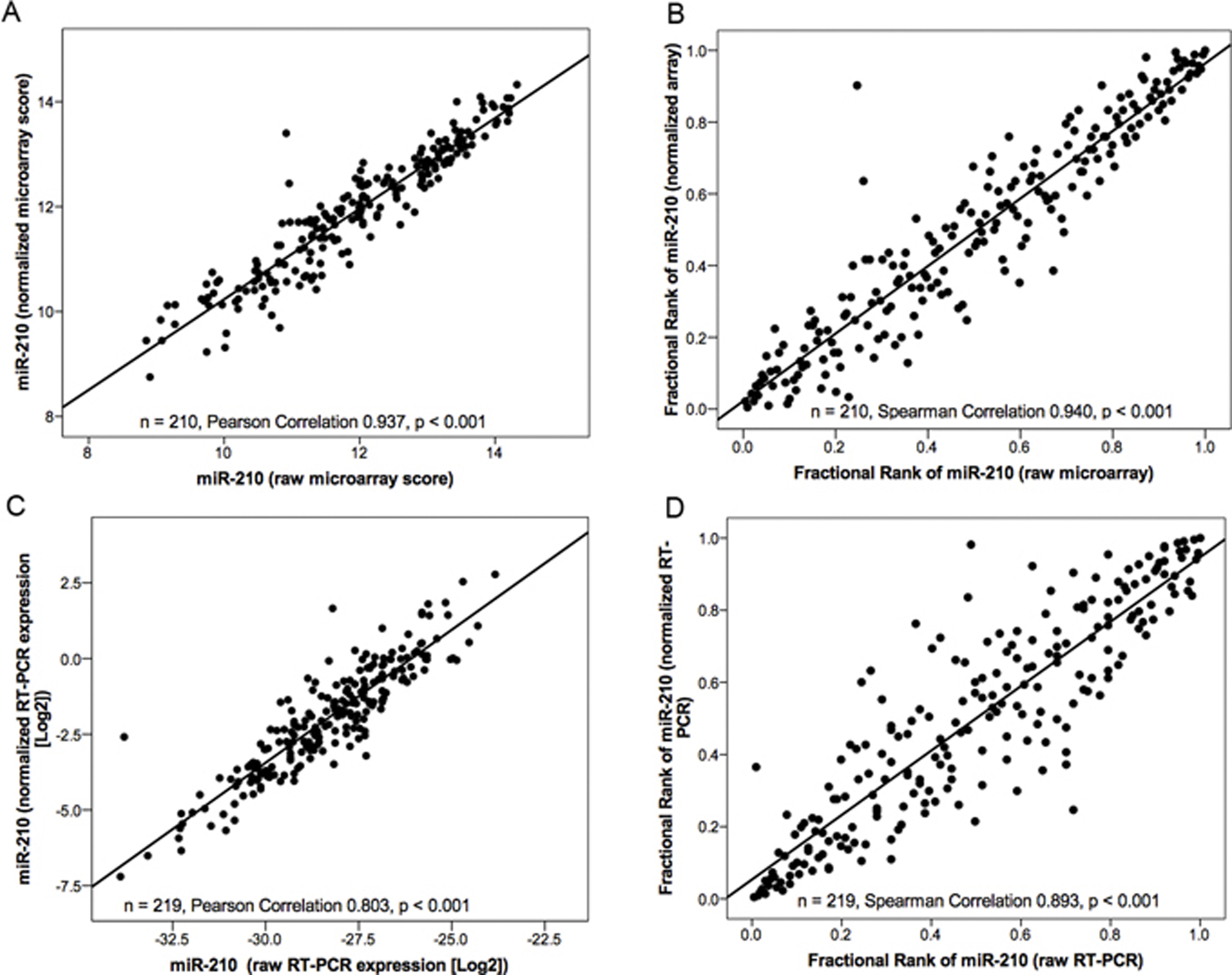

Supplement: Supplementary Figure S1 [file 6606076x1.tif]

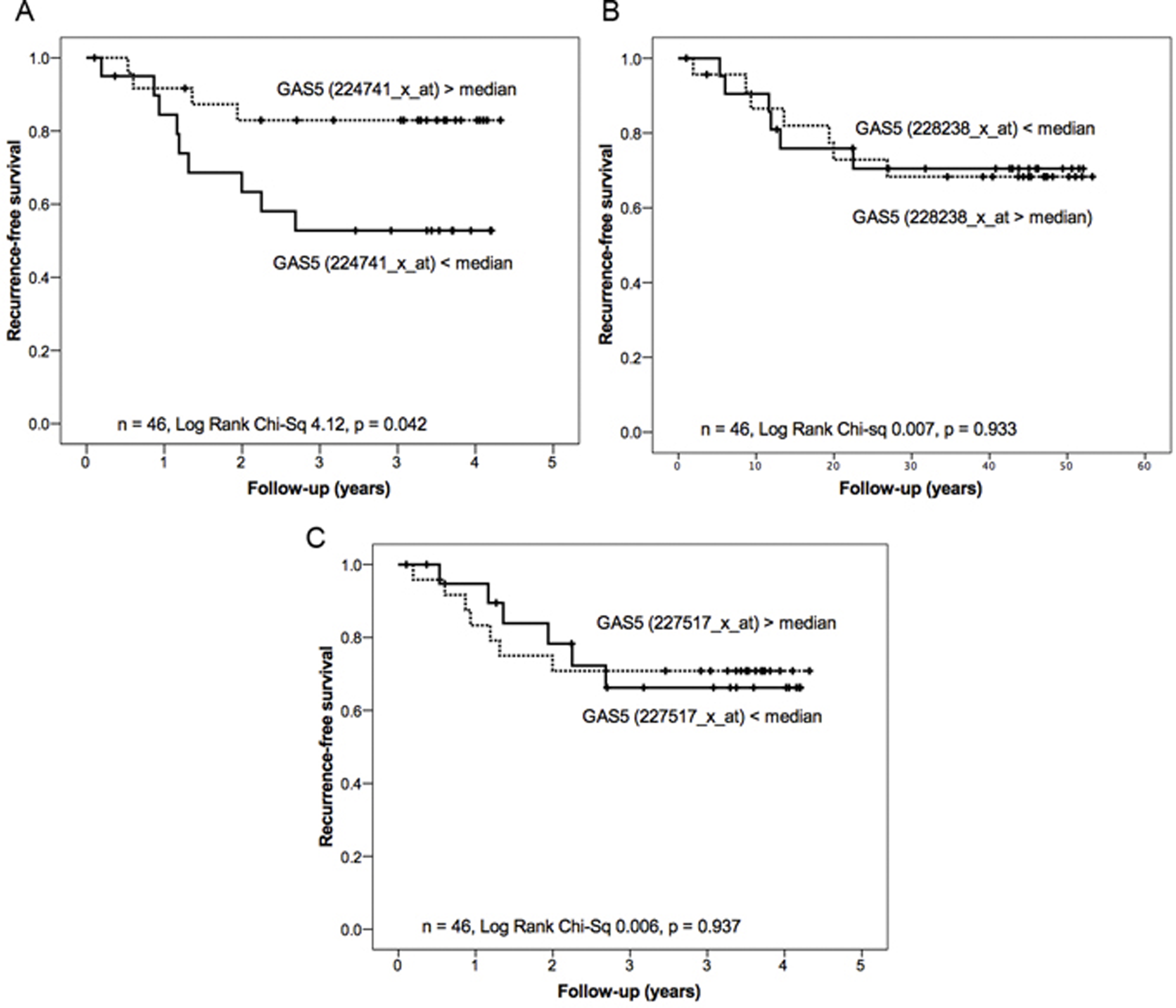

Supplement: Supplementary Figure S2 [file 6606076x2.tif]

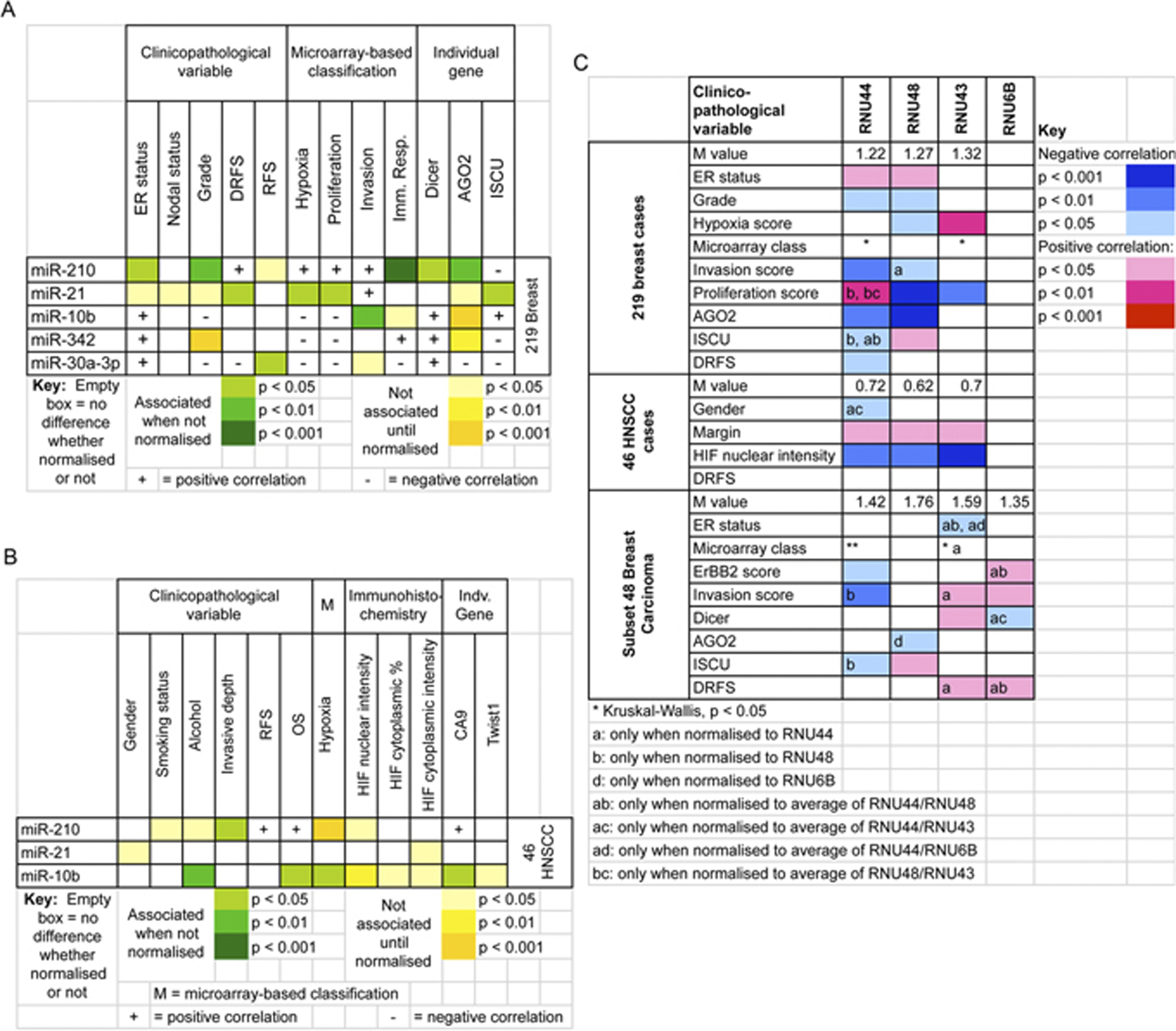

Supplement: Supplementary Figure S3 [file 6606076x3.tif]
