# Supplementary material for: The small-nucleolar RNAs commonly used for microRNA normalisation correlate with tumour pathology and prognosis
Source: Br J Cancer. 2011 Mar 15;104(7):1168–77. doi: 10.1038/sj.bjc.6606076 (PMC3068486; doi:10.1038/sj.bjc.6606076)
Supplement: Supplementary Tables and Figures Legends [file 6606076x4.doc]

Supplementary Table 1: Clinical details of patient series

| **Breast Cancer (Camps, 2008)** | |  | (n = 219) | |  |
| --- | --- | --- | --- | --- | --- |
| **Characteristic** | **Number** |  | **Characteristic** | | **Number** |
| **Age** |  |  | **Pathological LN status** | |  |
| Age (years) < 50 | 72 (67.1%) |  | N0 | | 127 (58%) |
| Age (years) ≥ 50 | 147 (32.9%) | | N+ | | 92 (42%) |
| Median (range) | 55 (26−80) |  |  | |  |
|  |  |  | **Histology** | |  |
| **Tumour size (cm)** |  |  | Infiltrating ductal | | 163 (74.4%) |
| <1.5 cm | 49 (22.4%) |  | Infiltrating lobular | | 27 (12.3%) |
| ≥ 1.5 cm | 170 (77.6%) | | Mixed | | 20 (9.1%) |
| Median (range) | 2.3 (0-9) |  | Others | | 9 (4.1%) |
|  |  |  |  | |  |
| **Grade** |  |  | **Molecular subtype** | |  |
| 1 | 42 (19.2%) |  | Basal | | 40 (18.3%) |
| 2 | 87 (40%) |  | ERRB2 | | 33 (15%) |
| 3 | 65 (29.4%) |  | LuminalA | | 78 (34.7%) |
| Missing | 25 (11.4%) |  | LuminalB | | 36 (16%) |
|  |  |  | Normal | | 29 (14%) |
| **ER status** |  |  | Missing | | 3 (2%) |
| Positive | 135 (61.7%) | |  | |  |
| Negative | 84 (38.3%) |  |  | |  |
|  |  |  |  | |  |
|  |  |  |  | |  |
|  |  |  |  | |  |
|  |  |  |  | |  |
|  |  |  |  | |  |
|  |  |  |  | |  |
|  |  |  |  | |  |
| **Subset of Camps 2008** | |  | (n = 48) | |  |
| Characteristic | **Number** |  | **Characteristic** | | **Number** |
| **Molecular subtype** |  |  | **ER status** | |  |
| Normal-like | 8 |  | Positive | | 23 (48%) |
| Luminal A | 8 |  | Negative | | 25 (52%) |
| Luminal B | 8 |  |  | |  |
| Basal | 16 |  |  | |  |
| ERBB2 | 8 |  |  | |  |
|  |  |  |  | |  |
|  |  |  |  | |  |
|  |  |  |  | |  |
|  |  |  |  | |  |
|  |  |  |  | |  |
| **HNSCC series (Winter, 2007)** | |  | (n = 46) | |  |
| **Characteristic** | **Number** |  | **Characteristic** | | **Number** |
| **Gender** |  |  | **Clinical follow-up (mths)** | | 41 (1-53) |
| Male | 37 (80%) |  | Disease-free survival | | 40 (2-53) |
| Female | 9 (20%) |  |  | |  |
|  |  |  | **Anatomic site** | |  |
| **Age** |  |  | Oral cavity | | 10 (22%) |
| Median (range) | 63 (43-92) |  | Oropharynx | | 21 (45%) |
|  |  |  | Hypopharynx | | 9 (20%) |
| **Post-operative therapy** |  |  | Larynx | | 5 (11%) |
| Radiotherapy | 41 (89%) |  | Paranasal sinus | | 1 (2%) |
| Chemotherapy | 3 (11%) |  |  | |  |
|  |  |  | **UICC stage** | |  |
| **Histological size** |  |  | I | | 2 (4%) |
| T1 | 5 (11%) |  | II | | 6 (13%) |
| T2 | 14 (30%) |  | III | | 3 (7%) |
| T3 | 7 (15%) |  | IV | | 35 (76%) |
| T4 | 20 (43%) |  |  | |  |
|  |  |  | **Smoking history** | |  |
| **Pathological LN status** |  |  | Never smoked | | 6 (13%) |
| N0 | 15 (33%) |  | Ex-smoker >1 year | | 12 (26%) |
| N+ | 31 (67%) |  | Current smoker | | 28 (61%) |
|  |  |  |  | |  |
| **Differentiation** |  |  | **Alcohol history** | |  |
| Well | 3 (6%) |  | Nil | | 10 (22%) |
| Moderately | 24 (52%) |  | Never heavy | | 14 (30%) |
| Poorly | 19 (41%) |  | Currently heavy | | 22 (48%) |
|  |  |  |  | |  |
| **Breast Cancer (part of Loi, 2008)** | | | (n = 152) | |  |
| **Characteristic** | **Number** |  | **Characteristic** | | **Number** |
| **Age** |  |  | **Grade** | |  |
| Age (years) < 50 | 40 (26.3%) |  | 1 | | 23 (15.1%) |
| Age (years) ≥ 50 | 112 (73.7%) | | 2 | | 74 (48.6%) |
| Median (range) | 61 (32−86) |  | 3 | | 32 (21%) |
|  |  |  | Missing | | 23 (15.1%) |
| **Tumour size (cm)** |  |  |  | |  |
| <1.5 cm | 27 (17.8) |  | **ER status** | |  |
| ≥ 1.5 cm | 125 (82.2) |  | Positive | | 122 (80%) |
| Median (range) | 2.1 (0.2-9) |  | Negative | | 26 (17%) |
|  |  |  | Missing | | 4 (2%) |
| **Pathological LN status** |  |  |  | |  |
| N0 | 120 (79%) |  |  | |  |
| N+ | 32 (21%) |  |  | |  |
| GEO number (GSE6532): Oxford samples | | |  | |  |
|  |  |  |  | |  |
| Note: series used here may differ slightly from original publication (1-2 cases) | | | | |  |
|  |  |  | |  |  |
| **Overlap between Camps and Loi** | | | | (n = 72) |  |
| **Characteristic** | **Number** |  | | **Characteristic** | **Number** |
| **Age** |  |  | | **Grade** |  |
| Age (years) < 50 | 21 (29.2%) |  | | 1 | 5 (6.9%) |
| Age (years) ≥ 50 | 51 (70.8%) |  | | 2 | 31 (43.1%) |
| Median (range) | 60 (32−79) |  | | 3 | 24 (33.3%) |
|  |  |  | | Missing | 12 (16.7%) |
| **Tumour size (cm)** |  |  | |  |  |
| <1.5 cm | 18 (75%) |  | | **ER status** |  |
| ≥ 1.5 cm | 54 (75%) |  | | Positive | 52 (72.2%) |
| Median (range) | 2.1 (0.7-9) |  | | Negative | 16 (22.2%) |
|  |  |  | | Missing | 4 (5.6%) |
| **Pathological LN status** |  |  | |  |  |
| N0 | 58 (80.6%) |  | |  |  |
| N+ | 14 (19.4%) |  | |  |  |

**Supplementary Table 2: Correlations between normalised and unnormalised RT-PCR and microarray data**


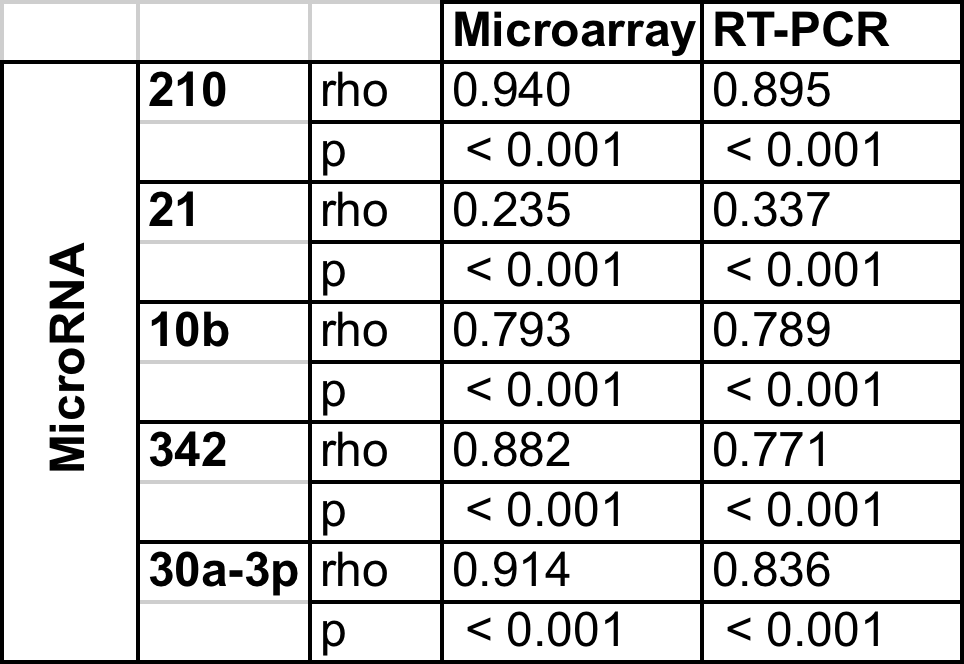


Note: rho = Spearman’s rho and p = 2-tailed significance, n = 210 for microarray, n = 219 for RT-PCR. Table shows correlation between raw and normalised data for miRNA, derived from microarray and RT-PCR data. For microarray data, data were normalised using overall mean expression value and adjusted for plate background. For RT-PCR, data were normalised to the median of 3 control genes RNU44, RNU43 and RNU48 using geNorm.

**Supplementary Table 3: Multivariate analysis of RNU44 in 219 Breast cases**

| **Variable** | **Significance** | **Hazard Ratio (HR)** | **95.0% CI for HR** | |
| --- | --- | --- | --- | --- |
|  |  |  | **Lower** | **Upper** |
| Estrogen receptor | .879 | 1.034 | .669 | 1.599 |
| Nodal status | .045 | 1.554 | 1.010 | 2.392 |
| Grade | .068 | 1.320 | .980 | 1.777 |
| Tumour size | .014 | 1.183 | 1.035 | 1.352 |
| RNU44 | .116 | .568 | .281 | 1.150 |

Note: Cox Regression for overall survival performed

**Supplementary Table 4: RPL3 correlation with RNU43 (RT-PCR)**

**
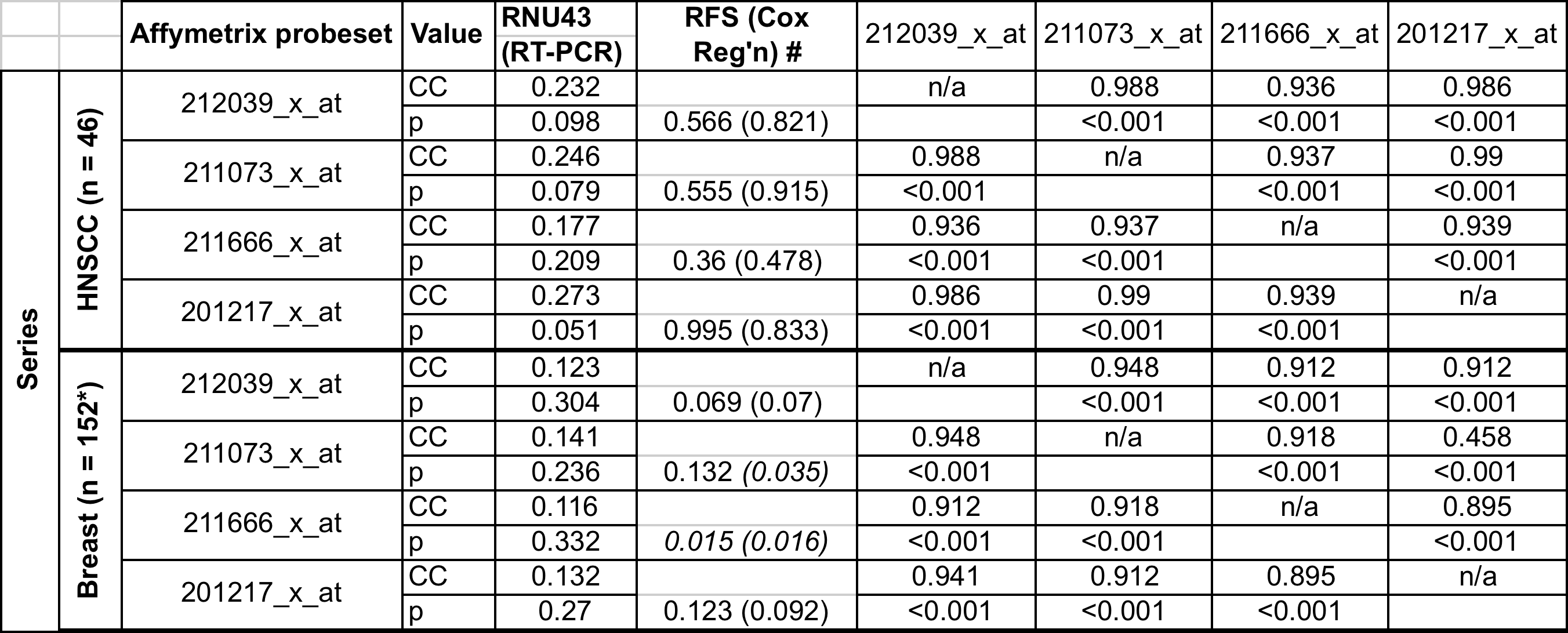
**

Note: CC = Correlation coefficient and p = 2-tailed significance

* Loi series – overlap of 72 cases with Camps series

# p-value, RFS = Recurrence-free survival Kaplan-Meier Log Rank; (Cox Regression)

### Supplementary Table 5: Association of miRNAs with distant recurrence-free survival, breast cancer

| **miRNA or control** | **0 controls** | **1 control gene** | **2 control genes** | **3 control genes** | **4 control genes** |
| --- | --- | --- | --- | --- | --- |
| miR-210 |  | U6***  43*  44**  48* | U6_43**  U6_44**  U6_48**  43_44**  43_48**  44_48* | U6_43_44***  U6_43_48**  U6_44_48**  43_44_48 ** |  |
| miR-21 |  | 44** | U6_44**  U6_48*  44_48* | U6_43_44*  U6_43_48*  U6_44_48**  43_44_48* |  |
| miR-10b |  |  |  |  |  |
| miR-342 |  |  |  |  |  |
| miR-30a-3p | *** | U6**  44* | U6_48*  43_44*  43_48* | U6_43_44*  U6_43_48*  43_44_48* |  |

***Notes:***

Table shows p-value for association with prognosis (if any) of 5 miRNA, normalised to 1, 2, 3 or 4 control genes. Red box – positive association, ie higher level associated with a worse prognosis; Blue box – negative association (Kaplan-Meier). miRNA and snoRNA measured by RT-PCR in 48 cases of breast cancer. Control genes are indicated by number (43 – RNU43, 44 – RNU44, 48 – RNU48, U6 – RNU6B). * p < 0.05, ** p < 0.01, *** p < 0.001.

**Figure Legends**

**Supplementary Figure 1:** **Values of miRNA from RT-PCR are noisier than from microarray.** (A) Scatter plot showing values for miR-210 derived from raw microarray values (x-axis) against values for miR-210 once normalised (using overall mean expression value, quantile normalisation, and adjusting for plate background, y-axis) in the breast cancer series. (B) Scatterplot showing values in (A) converted to fractional rank (between 0-1). (C) Scatter plot showing relative unnormalised expression of miR-210 measured by RT-PCR (x-axis) for the same series, compared with relative expression of miR-210 normalised to 3 control genes RNU44, RNU43 and RNU48. (D) Same values as (C) converted to fractional rank.

**Supplementary Figure 2:** GAS5 probes and prognosis. Kaplan-Meier curve of recurrence-free survival for patients with HNSCC stratified according to relative expression of GAS5 measured by microarray (3 probesets shown). Expression levels are stratified by median value.

**Supplementary Figure 3:** Alternative versions of Fig 2A, 2B, and 3A, showing the p – values. (A) and (B) Association between miRNA and clinicopathological factors can be over- or underestimated depending on the interaction with snoRNA control. miRNA and snoRNA measured by RT-PCR in 219 breast cancers (A), 46 HNSCCs (B). Background colour of box indicates type of misassociation: green boxes – miRNA associated with factor before it is normalised to snoRNA; yellow boxes – miRNA not associated until normalised; white boxes – consistent association between miRNA and factor whether normalised or not; +/- show direction of statistically significant correlation (if any). (C) Heat map showing direct association or correlation between snoRNAs (measured by RT-PCR) and clinicopathological factors. Blue background – negative correlation; pink background – positive correlation; white background – no correlation, except * (significant association with molecular subtype). ER status = estrogen receptor status, nodal status = binary; smoking/alcohol status = never, ex-user, current user. DRFS or RFS: distant recurrence-free survival or recurrence-free survival (STEEP criteria), OS – overall survival; expression stratified by median value, positive correlation = higher level associated with poorer prognosis. Details of microarray based classifications including hypoxia metagene score, proliferation score, invasion score, immune response score (Imm. Resp.), and scoring system for immunohistochemistry, in methods section. AGO2 = EIF2C2, (Argonaute 2), ISCU = Iron-Sulfur Cluster Homologue (E. coli), CA9 = carbonic anhydrase 9. M value – gene stability measure, defined as average pair wise variation of a particular gene with all other control genes, measured by geNorm algorithm. Results shown for Spearman correlation (continuous variables), Mann-Whitney (2 categorical variables), Kruskal-Wallis (>2 categorical variables).
